# Large animal models for translational research in acute kidney injury

**DOI:** 10.1080/0886022X.2020.1830108

**Published:** 2020-10-12

**Authors:** Balamurugan Packialakshmi, Ian J. Stewart, David M. Burmeister, Kevin K. Chung, Xiaoming Zhou

**Affiliations:** Department of Medicine, Uniformed Services University of the Health Sciences, Bethesda, MD, USA

**Keywords:** Immune response, innate immunity, adaptive immunity, ischemia-reperfusion, cisplatin, swine

## Abstract

While extensive research using animal models has improved the understanding of acute kidney injury (AKI), this knowledge has not been translated into effective treatments. Many promising interventions for AKI identified in mice and rats have not been validated in subsequent clinical trials. As a result, the mortality rate of AKI patients remains high. Inflammation plays a fundamental role in the pathogenesis of AKI, and one reason for the failure to translate promising therapeutics may lie in the profound difference between the immune systems of rodents and humans. The immune systems of large animals such as swine, nonhuman primates, sheep, dogs and cats, more closely resemble the human immune system. Therefore, in the absence of a basic understanding of the pathophysiology of human AKI, large animals are attractive models to test novel interventions. However, there is a lack of reviews on large animal models for AKI in the literature. In this review, we will first highlight differences in innate and adaptive immunities among rodents, large animals, and humans in relation to AKI. After illustrating the potential merits of large animals in testing therapies for AKI, we will summarize the current state of the evidence in terms of what therapeutics have been tested in large animal models. The aim of this review is not to suggest that murine models are not valid to study AKI. Instead, our objective is to demonstrate that large animal models can serve as valuable and complementary tools in translating potential therapeutics into clinical practice.

## Introduction

Acute kidney injury (AKI) is defined by a sudden reduction in renal function and is associated with mortality in observational studies. For example, one study of combat casualties found that AKI was independently associated with mortality after adjustment for demographics, hemodynamics, and injury severity [1]. Furthermore, AKI has been associated with long-term complications to include chronic kidney disease [2], end stage kidney disease [2], hypertension [3,4], heart failure [5,6], and mortality [7,8]. Both local and systemic inflammation plays an essential role in the pathogenesis of AKI. Despite extensive research and significant progress made in understanding basic mechanisms for the disease in rodent models, no clinically proven interventions exist to prevent AKI, accelerate recovery of AKI, or reduce progression of AKI to CKD in patients [9].

Owing to their ease of handling, breeding, and genetic modification, mice and rats are often chosen to study the basic mechanisms of AKI and to evaluate potential therapeutics. However, many promising interventions for AKI found in mice and rats have not been reproduced in clinical trials. For example, N-acetylcysteine has been demonstrated to prevent AKI induced by ischemia/reperfusion (I/R) [10], sepsis [11], rhabdomyolysis [12], and contrast medium [13] in rodents, but failed to prevent AKI induced by contrast [14], vancomycin [15], and I/R following cardiac surgery [16–18] in clinical trials. Many reasons are postulated for the failure to transition therapeutics from animal models to clinical practice [19,20], but one factor is that rodents and humans have developed different innate and adaptive immune systems, since they diverged somewhere between 65 and 75 million years ago. For instance, humans have 50–70% neutrophils and 20–40% lymphocytes, whereas C57BL/6 mice contain only 10–25% neutrophils, approximate 2% monocytes and 75–90% lymphocytes [21].

In the absence of a basic understanding of the molecular pathophysiology of human AKI, large animal models (LAMs) such as porcine, simian, ovine, canine, and feline models are attractive to test novel therapies, because their immune systems more closely resemble the human’s when compared to rodents [22–25]. The FDA recommends that an intervention be tested in more than one animal models before the submission of an investigative new drug application (https://www.fda.gov/drugs/drug-development-tool-ddt-qualification-programs/animal-model-qualification-amqp-program). It is a preferred practice that at least one of these models be conducted in large animals. There are many excellent reviews of therapies tested for AKI in mice and rats in the literature. However, there is a lack of such reviews in LAMs. This review will first focus on the differences in innate and adaptive immunities among rodents, large animals, and humans in relation to AKI. After illustrating the value of LAM in testing potential therapies for AKI, this review will then summarize what has been learned from such models to date.

## Overview of molecular mechanisms of AKI

The most common cause of AKI is ischemia–reperfusion injury (IRI) induced by transplantation, trauma, burns, or sepsis, that leads to a reduction of renal blood flow. The second most common cause is certain medications and toxins [26,27]. Regardless of the initial insult, however, these different causes share some common pathophysiology. While the exact sequence of events can differ, the pathophysiology includes inflammation, immune damage, oxidative stress, reduction in renal perfusion, and both apoptotic and necrotic cell death [28–31]. Following the insult, damaged cells in the kidney and/or other tissues release danger-associated molecular patterns (DAMPs), such as high mobility group box-1, S100A8, fibronectin, and DNA. In the case of sepsis-induced AKI, pathogens release pathogen-associated molecular patterns (PAMPs) such as lipopolysaccharides (LPS). The released DAMPs and PAMPs then bind pattern recognition receptors (PRR), including Toll-like receptors (TLR), on the surface of kidney cells and leukocytes and activate NF-κB and other pro-inflammatory transcription factors. This results in the release of cytokines and chemokines with subsequent adhesion and infiltration of leukocytes into the renal parenchyma, culminating in endothelial dysfunction, impaired mitochondrial function, disturbed redox balance, epithelial apoptosis, and necrosis. After these pathologic changes reach a threshold, kidney function is compromised as evidenced clinically by oliguria as well as increases in serum creatinine and blood urea nitrogen [28,31–33].

## The differences in innate immunity among rodents, large animals, and humans

Given the acute nature of AKI, innate immunity is a predominant driving factor in its pathophysiology. Innate immunity is composed of soluble molecule-mediated immunity and cell-mediated immunity. The former includes complement, cytokines, and chemokines, while the latter includes macrophages, neutrophils, natural killer, and dendritic cells [34,35]. The infiltration of innate immune cells into the kidney is coordinated by a large array of chemokines. It has been reported that the chemokines CCL24/CCL26, CXCL8/IL-8, CXCL7, CXCL11, CCL13, CCL14, CCL15, CCL18, and CCL23 and are present in humans, but not in mice. Conversely, CCL12, CCL6, CCL9, and CXCL15 have been identified in mice, but not in humans [21]. Some of these chemokines have been demonstrated to be important for the development of AKI. For example, CCL24/CCL26 is increased in patients with subclinical and clinical rejection of kidney allograft in patients [36].

Neutrophils are the most abundant type of granulocytes and make up 40–70% of all white blood cells in humans. They are also the most abundant leukocytes infiltrating the kidney immediately after IRI [37]. Neutrophils produce and secrete cytotoxic compounds such as reactive oxygen species, while adhering to the endothelium and extravagating into the affected renal tissue. CXCL8/IL-8 is the primary chemoattractant for human neutrophil recruitment. Serum CXCL8/IL-8 levels predict AKI in patients with acute pancreatitis [38], after cardiac surgery [39] and liver transplantation [40]. Conversely, CCL12 probably mediates tubular regeneration and functional recovery from cisplatin-induced AKI following inhibition of dipeptidyl peptidase-4 in a murine model [41]. Given the important roles that these molecular pathways play in the development of AKI, it becomes apparent that immunomodulatory findings from rodent AKI models may have certain limitations [19].

Macrophages are the most abundant immune cells within the kidney, but monocytes are rare in the healthy kidney. After recruitment into the injured kidney, monocytes differentiate into proinflammatory M1 and/or immune-regulatory M2 types of macrophages [42]. Although the exact stimuli and mechanisms causing differentiation into M1 versus M2 macrophages remain unclear, binding of DAMPs and PAMPs to PRR induces activation of M1 macrophages from both resident and differentiated macrophages. Activated M1 macrophages then secrete proinflammatory cytokines such as IL-1β, IL-6, IL-12, IL-18, and TNF-α, and release reactive oxygen and nitrogen species, which contribute to AKI. M2 macrophages release anti-inflammatory mediators such as IL-10 and TGF-β, limiting inflammatory responses in the kidney. Inducible nitric oxide synthase (iNOS) is the primary source of reactive nitrogen species in macrophages in mice [34,43]. In murine macrophages, iNOS is up-regulated by several orders of magnitude upon incubation with IFN-γ or LPS. In contrast, iNOS is not generally present in human macrophages; although there are some reports showing expression of iNOS under severe disease conditions [44]. Instead, IFN-γ and LPS stimulate indoleamine 2,3-dioxygenase (IDO), an anti-inflammatory enzyme, in human macrophages [44]. Furthermore, the lethal dose of LPS for mice is about 1000 times higher than that for humans [44].

These differences must be taken into consideration when translating potential therapeutic candidates that target macrophages which have been identified in murine models. For example, Rasburicase prevents cisplatin-induced AKI in rats in part through reduction of macrophage infiltration [45]. However, it failed to prevent AKI after cardiovascular surgery in patients [46]. Probiotics ameliorate I/R-induced AKI by increasing M2 macrophages in rats [47]. However, it has been demonstrated that the density of CD163^+^ M2 macrophages in the human kidney correlates with the severity of a variety of renal diseases, including: AKI [48], acute tubular injury [49], IgA nephropathy [50], chronic kidney allograft injury [51], and lupus nephritis [52]. Moreover, erythropoietin prevents AKI in various murine models in part by reducing the infiltration of macrophages and promoting M2 macrophage phenotype [20,53]. However, it has failed in the majority of clinical studies [20,44].

**Figure 1.**
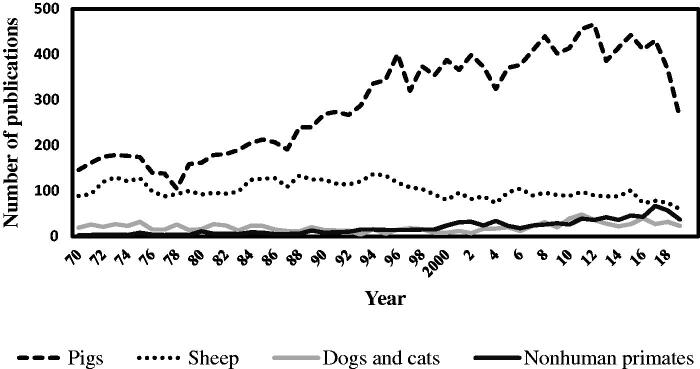
Large animal models in kidney research indexed in the PubMed from 1970 to 2019. (Year X-axis) retrieved using a search query: (‘renal’ OR ‘kidney’) AND (‘species name’) on May 20, 2020. The overall trend shows the preference for porcine models.

In contrast to rodents, the immune systems of large animal animals are more analogous to humans. Similar to humans, dogs and pigs have intralobular lymphatics, which is absent or not yet defined in mice [54]. Among LAM, pigs are the most popular large animals used in the kidney research (Figure 1). Pigs share more than 80% of immune parameters with humans as compared to mice that share less than 10% [22,23,55]. Similar to humans, and in contrast to mice, pigs have a high percentage of neutrophils in the peripheral blood (50–70%), express CXCL8/IL-8, do not express iNOS in macrophages, IFN-γ and LPS stimulate IDO in macrophages, and pigs are sensitive to endotoxin shock [23,56]. As in humans, expression of CXCL-8/IL-8 and infiltration of M2 macrophages correlate with disease severity in pigs. This is evidenced by studies in swine showing that attenuation of burn- and trauma-induced AKI by oral resuscitation and vitamin C is associated with a reduction of serum levels of CXCL-8/IL-8 [57,58] and that mitigation of I/R-induced AKI by inhibiting complement pathway is associated with decrease of infiltration of CD163^+^ M2 macrophages into the kidney [59].

TLR4, the receptor for LPS, is arguably the most important receptor of PRR. Swine and human TLR4 genes have approximately 83% sequence homology in three exon sequences as opposed to 75% between humans and mice. Overall, the porcine TLR4 promoter shares more features with the human TLR4 promoter than its murine counterpart [60]. Moreover, porcine and human kidneys express similar level of TLR4 protein, whereas the murine kidney expresses twice as much [61]. TLR signaling is MyD88-dependent with the exception of TLR3. The putative porcine MyD88 protein shares a higher level of homology with its human (87.2% amino acid identity) than with its mouse (77.4% amino acid identity) counterpart [62].

In terms of nonhuman primates (NHPs), comparison of the chimpanzee and human genomes has revealed remarkable conservation of genes; about 30% are identical and single base pair substitutions account for about half of the genetic change [63]. Chimpanzee and human TLR4 gene sequences only have differences at three amino acid positions [24,25]. Features of the innate immune system are not where the similarities stop between large animals and humans, but these commonalities also extend to the adaptive immune system.

## The differences in adaptive immunity among rodents, large animals, and humans

Adaptive immunity is mediated by T and B lymphocytes. Although there is some discrepancy, the majority of studies have demonstrated or suggested that CD4^+^ T cells are a factor in driving the initial phase of AKI in murine models, whereas the roles of cytotoxic T cells and B cells remain unclear [64]. Whether CD4^+^ T cells are involved in human AKI is unknown, but in pig models of renal autotransplantation, addition of polyethylene glycol, trimetazidine, and/or an inhibitor of complement to preservation solutions decreases infiltration of macrophages and T or CD4^+^ T cells into the kidney, and results in reduced graft injury [65–68]. Depending on inducers, CD4^+^ T cells can be activated and differentiated into different subsets including pro-inflammatory Th1 and Th17 cells and regulatory T cells (Treg) [64]. In rodents, both Th1 and Th17 subsets contribute to AKI through secreting pro-inflammatory cytokines and recruiting other types of pro-inflammatory cells into the kidney, whereas Treg cells protect the kidney from IRI and help resolution of AKI by limiting inflammation [64,69,70].

An important difference in CD4^+^ T cell activation exists between mice and humans. In order to fully activate CD4^+^ T cells, the costimulatory receptor CD28 has to be activated. Nearly all mouse CD4^+^ T cells express CD28, whereas only 80% of human CD4^+^ T cells express CD28 on their surface [21]. Several studies have shown that blocking the CD28 costimulatory pathway with CTLA-4 Ig decreases infiltration of monocytes and ameliorates I/R-induced AKI in rodents [71–73]. However, CTLA-4 Ig does not significantly affect human Th1 cells in renal graft tissue [74]. Moreover, immunotherapy with CTLA-4 antibodies causes AKI in some cancer patients [75,76].

Differences also exist at the differentiation and phenotypic levels between rodent and human T cells. In humans, type I interferon IFN-α stimulates Th1 differentiation, whereas it does not in mice [21]. The human Th17 cells express surface CCR2, whereas mouse Th17 cells express CCR7 instead [77]. Human Th17 cells have multiple subsets, whether similar subsets exist in rodents is not clear [78,79]. Mouse immune tolerance is a poor predictor of human tolerance [80]. However, this gap can be alleviated by NHP Treg cells, which display phenotypic and functional similarities with human Treg cells [81]. Taken together, differences in immunology between humans and mice may help explain the lack of successful pharmacotherapies for AKI in patients.

## The differences in gut microbiota among rodents, large animals, and humans

Gut microbiota regulates immune responses in pre-clinical models and humans [82,83]. Emerging evidence indicates that gut microbiota also plays a critical role in modulating various renal diseases [84,85]. The majority of our current knowledge of the effects of gut microbiota on AKI is from studies using mice and rats, and data from LAM studies are scarce [85–87]. There is a positive correlation between gut *Rothia* and *Staphylococcus* levels and renal function in a rat I/R AKI model [88]. Depending on experimental contexts, both reno-protective and reno-harmful effects of gut microbiota have been reported. Germ-free mice have an unexpectedly high frequency of natural killer T cells and abundant T cells in the kidney and are prone to I/R-induced AKI [64]. In contrary, depletion of mouse gut microbiota by an antibiotic-cocktail significantly attenuates I/R-induced AKI associated with low expression of F4/80 macrophages and pro-inflammatory chemokines [89]. Intestinal microbiota modulates immune reactions in AKI through their metabolites such as short chain fatty acids, trimethylamine-N-Oxide, and D-amino acids [86,90]. While it is known that there is a gut-kidney cross talk, it has only recently been demonstrated that AKI can influence intestinal microbiomes. Traditionally, I/R-induced AKI has been regarded as sterile inflammation. However, Yang *et al* recently reported that I/R-induced AKI provokes intestinal dysbiosis and bacterial translocation which is associated with increases of Th1 and Th17 responses as well as activation of neutrophils and M1 macrophages in a mouse model. It is possible that the translocation of gut resident bacteria exacerbates inflammatory process in I/R-induced AKI [91].

Because gut microbiota modulate immune responses in AKI, manipulating the gut microbiota has been proposed as a possible new therapeutic avenue to treat AKI. D-serine, a gut microbiota metabolite which is decreased in AKI patients, mitigates I/R-induced AKI in mice when given orally [90]. Anticipating further studies in this area, it is important to recognize the differences amongst murine, large animal, and human gut microbiota [92]. Although mice and humans share considerable anatomical, histological, and physiological similarities of the intestinal tract, the differences in the size of intestinal tract and dietary habits contribute to only 4% of shared bacterial genes in mice and humans [93]. For example, mice harbor segmented filamentous bacteria, which have a profound effect on the maturation of innate immune system, whereas these bacteria have not been identified in human adults [93]. On the other hand, *Macaca fascicularis* shares 39.49% of gut microbiome genes with humans and 25.45% of the genes with pigs; this is compared with only 0.6% of the genes with mice [94]. Like humans, the pig gut microbiota mainly consists of Formicates and Bacteroidetes phyla [95]. Further, human microbiota-associated piglets have been established by inoculating microbiota from infants, children, and adults. These piglets share even more gut microbiota with humans than conventional piglets [96]. Since gut microbiota regulate immune responses, the differences in murine and human gut microbiota compositions could contribute to the differences in immune responses seen during AKI. Likewise, similarities between large animal and human microbiota may explain some of the shared characteristics in AKI-induced immune responses.

## Mechanistic strategies of AKI for evaluating therapeutics in LAM

The strategies that have been evaluated to intervene on AKI in LAM are similar to those evaluated in mice and rats [30]. They include limiting inflammation, reducing oxidative stress, increasing renal blood flow, and stem cell therapy. Multiple approaches targeting various steps in immune pathways have shown promising results in LAM (Tables 1–4). They include inhibition of TNF-α by an antibody in NHP [165] and the chemical FR167653 in pigs [112], infiltration of macrophages, and T-helper cells by the chemical TBC-1269 in pigs [105], and complement cascade by compstatin CP40 in cynomolgus monkeys [153]. Erythropoietin is well known for its erythropoietic effect and was later recognized to be an anti-inflammatory cytokine. Erythropoietin acutely improves glomerular filtration rate (GFR) in pigs after IRI and reduces both renal and circulating levels of TNF-α [106]. Erythropoietin also shows a preventive effect against AKI in NHP [156]. The side effect of increased red blood cells, and higher levels of hematocrit, can be minimized by an 11 amino acid nonerythropoietic peptide (ARA290) without losing anti-inflammatory properties [109]. Further, ARA290 reduces interstitial fibrosis by decreasing α-smooth muscle actin in the porcine kidney [109]. However, only two out of nine clinical trials found the preventive effect of erythropoietin against I/R-induced AKI in patients, indicating that even LAM is not a complete replica of humans in the translational research [20].

**Table 1.** Major therapeutic approaches tested in porcine models.

Model	Therapy	Therapeutic target	References
Ischemia Reperfusion	Doxycycline	MMP inhibition reduces lipid peroxidation	[97]
	Carbon monoxide inhalation	Activates HSP70 response, anti-apoptosis, anti-inflammatory	[98]
	Sitaxentan	Endothelin-A-receptor antagonist improves hypoxia	[99]
	Sildenafil citrate	Increases NO bioavailability and reduces inflammation	[100]
	Ulinastatin	Antioxidative stress, anti-inflammatory	[101,102]
	Alkaline phosphatase	Dephosphorylation of signaling molecules, adenine, etc.	[103]
	Canrenoate	Reduces oxidative stress	[104]
	TBC-1269	Selectin ligand blockade prevents leucocyte adhesion	[105]
	Erythropoietin	Anti-inflammatory decreases TNF-α	[106]
	Erythropoietin	Reduces noradrenaline requirements to achieve the hemodynamic targets	[107]
	Carbamylated erythropoietin or recombinant human erythropoietin	Failed to attenuate prolonged ischemia-induced AKI	[108]
	ARA290, EPO derivative	Reduces MCP-1 and IL-6 and interstitial fibrosis	[109]
	Cyclic helix B peptide	Antiapoptosis, tissue protection	[110]
	AP214	α-MSH analogue, anti-inflammatory	[111]
	rhC1 inhibitor	Inhibits complement system	[59]
	FR167653	P-38 MAPK inhibitor reduces TNF-α	[112,113,114]
	Anti-high-mobility group box 1 antibody	Reduces blood inflammatory cytokine levels	[115]
	Caspase-3 siRNA	Inhibits apoptosis	[116]
	hemoreperfusion with leucocyte-depleted blood	Inhibits inflammation and apoptosis	[117]
	Hydrogen or sodium sulfide	Reduces oxidative stress, inflammatory cytokines, iNOS	[118,119]
	Hydrogen gas	No effect	[120]
	Anti-CD47 antibody	Reduces inflammation and apoptosis	[121]
	Atrial natriuretic peptide	Improves blood flow to the kidneys	[122]
	Fenoldopam	Dopamine D1 receptor agonist improves blood flow	[123]
	NO + corticosteroids	Reduces vascular resistance and inflammation	[124]
	Trimetazidine	Inhibits mitochondria oxidation of fatty acids	[125]
	Vitamins C and E	Antioxidant and nutrients	[58,126]
	Mesenchymal stem cells	Decreases inflammation, oxidative stress, and fibrosis	[127,128–130]
	Mitochondrial transplantation	Reduces IL-6 expression in the renal cortex	[131]
	Meclizine	Up-regulates glycolysis and reduces oxidative stress	[132]
	N-acetylcysteine	Reduces oxidative stress and improves hemodynamics	[133]
	N-acetylcysteine	Reduces oxidative stress	[134]
	Ascorbic acid + selenium + tocoferol and N-acetyl-cysteine	No effect on oxidative stress	[135]
	Resveratrol	Decreases oxidative stress and apoptosis	[136]
	Elamipretide	Improves mitochondrial function	[137]
	Danegaptide (not effective)	Targets mitochondrial Connexin 43 channels	[138]
	TRVP channel inhibitor	Increases blood flow to the kidneys	[139]
	Calcitonin antibodies	Blocks the effect of calcitonin	
Drugs (nephrotoxins)	Rapamycin	Autophagy, mitophagy, reduces ROS	[140]
	Magnesium	Improves renal function	[141]
	Retinoic acid	Autophagy activation and apoptosis inhibition	[142]
	microRNA-30c (miR-30c)	Reduces the inflammation by targeting NLRP3 inflammasome	[143]
	N-acetylcysteine	Reduces oxidative damage and enhances autophage	[144]
	Inhaled nitric oxide	Vasodialation, reduces inflammation, counteracts prostanoid pathways	[145]
Sepsis	Calcitonin antibody	Blocks calcitonin action	[146]
	Erythropoietin	No effect	[147]
	Polymyxin B hemoperfusion	Neutralize LPS	[148]
	Peritoneal negative pressure	Inhibits inflammation	[149]
Burn	Enteral fluid resuscitation	Useful in a resource poor environment, reduces circulating cytokines	[150]
Hemorrhagic shock	Aggressive care (AC)	Several treatments are combined together	[151]
	Terlipressin	Vasopressin analog improves blood pressure, reduces necrosis	[152]

**Table 2.** Major therapeutic approaches tested in NHP models.

Model	Therapy	Therapeutic target	Ref
Surgery and hemorrhage shock	Compstatin CP40	Complement protein C3 inhibition	[153,154]
	Artificial support to liver and kidney	Alternative to renal replacement therapy	[155]
Ischemia/reperfusion	Erythropoietin	Anti-inflammatory	[156]
	FR260330	Inhibits iNOS and inflammation	[157]
	Monoclonal antibody mAb107	inhibits proinflammatory integrin CD11b/CD18 to prevent progression AKI to CKD	[158]
	Mesenchymal stem cells therapy	Paracrine effects, trans differentiation	[159]
Drugs (e.g. Cisplatin gentamycin)	Mesenchymal stem cells therapy	Repair and renewal of cells in the kidney	[160]
	biomarkers	Not for therapy but to explore biomarkers	[161–[162]
Sepsis	Chimeric antibody against Factor-X	Block the coagulation cascade	[163]
	Cell-permeable peptide (TVP)	Degrades pathogenic toxins in lysosomes	[164]
	Anti-TNF-α antibody	Anti-inflammatory and reduces coagulation	[165]
	Fondaparinux pentasaccharide	Anticoagulant, inhibits factor Xa	[166]
	Diethylenetriamine pentaacetic acid	Chelates iron and reduces oxidative radicals	[167]

**Table 3.** Major therapeutic approaches tested in ovines.

Model	Therapy	Therapeutic target	Ref
Sepsis	TAK-242 inhibitor of Toll like receptor 4 (TLR4)	Interrupts LPS activation	[168]
	Dexmedetomidine, α2-adrenergic receptor agonist	adjunct therapy to norepinephrine infusion, reduces IL-6, increases IL-10	[169]
	Furosemide	Diuretic increases sodium excretion, decreases oxygen consumption	[170]
	Arginine vasopressin (AVP) and norepinephrine (NE)	Improves blood pressure and renal blood flow	[171,172]
	Angiotensin II	Vasoconstrictor improves blood flow and creatinine clearance	[173]
	Various resuscitation fluids	Improves blood volume and pressure	[174–175]
IRI	Ketamine, NMDA receptor antagonists	Reduces inflammation, macrophages infiltration	[176]
	organic mononitrites of 1,2-propanediol (PDNO)	Vasodilator, improves oxygen utilization in kidneys	[177]
	Zinc	Cyto-protective, upregulates hypoxia inducible factor proteins, not clear	[178]
	Mesenchymal stem cells	Not effective in the sheep model	[179]
Cardiopulmonary bypass	Metaraminol, α1-adrenergic receptor agonist	Improves oxygenation in renal medulla	[180]
Hemorrhage shock	7.5% NaCI/6% Dextran-70 (HSD) as resuscitation fluid	Improves plasma volume, hemodynamics and safe during dehydration	[181]

**Table 4.** Major therapeutic approaches tested in felines and canines.

Model	Animal	Therapy	Therapeutic target	Ref
Sepsis	Dogs	Recombinant human brain natriuretic peptide (rhBNP)	Improves renal blood flow by NO generation	[182]
IRI	Cats	Mesenchymal stem cell therapy	No therapeutic effects	[183]
	Dogs	CRRL269, guanylyl cyclase A receptor peptide activator	Reduces apoptosis, modulation of intracellular Ca2+ levels	[184]
	Dogs	Vitamin C	Antioxidant, reduces BUN values, not completely effective	[185]
	Dogs	Sildenafil	Anti-inflammatory, antioxidant and anti-apoptotic	[186]
	Dogs	Mesenchymal stem cells therapy	Repair the renal tissues	[187]
	Dogs	combination therapy (n-acetyl cysteine (NAC) +sodium nitroprusside (SNP) + phosphormidon)	Antioxidant, vasodilator, endothelin inhibitor	[188]
	Dogs	Prostaglandin E2 (PGE2)	Creatinine and urea clearances were improved but the exact mechanism is not clear	[189]
	Dogs	ATP-MgCl_2_	Improves energy metabolism, claimed to be useful in humans also	[190]
Drug	Dogs	ATP-MgCl_2_	Worsens the renal parameters not useful, contradicts the IRI model results	[191]
	Dogs	Atrial natriuretic factor (ANF)	Increases and maintains GFR	[192]
Hemorrhage shock	Dogs	Fenoldopam	Dopamine D1 receptor agonist, improves blood flow	[193]

Strategies that target the underlying cause of AKI may also be effective in inhibiting inflammation, which can potentially be discovered by the use of LAM. For example, an exciting recent avenue of interest in the trauma field involves the role of endotheliopathy in inflammatory processes [194,195]. This is mediated in part by glycocalyx shedding which predisposes the vasculature to cellular extravasation into the interstitial space. This has been examined in models of both cardiopulmonary bypass [196] and burns [197], as both renal and circulating markers of glycocalyx shedding (e.g. syndecan) are implicated in the development of AKI. In the context of burn trauma, it is known that resuscitation fluid can affect the degree of endothelial dysfunction [198]. However, it is also known that hypovolemia plays a role in burn-induced AKI, and fluid resuscitation ameliorates burn-induced AKI and is associated with decreases in IL-1β, IL-6, IFN-γ, and GM-CSF in pigs [150].

Oxidative and nitrostative stresses also mediate AKI. Sodium sulfides or hydrogen sulfide attenuate I/R-induced AKI in a porcine model by reducing nitrostative stress, lipid peroxidation, IL-1β, and IL-6 [118]. Rapamycin mitigates AKI by reducing lipid peroxidation, protein carbonylation, NF-κB and by promoting mitophagy in a mini-pig model [140]. Mitochondria are a major source of reactive oxygen species which may be ameliorated by Elamipretide, which is a mitochondria-targeted tetrapeptide. By decreasing ROS generation and stabilizing cardiolipin [199], an important component of the inner mitochondrial membrane, Elamipretide improves renal function in atherosclerotic renal artery stenosis-induced injury in domestic pigs [137].

Increase of blood flow to the kidney by vasodilation through increasing either nitric oxide bioavailability, endothelin inhibition, or administration of hormone and neurotransmitter analogues has been shown to ameliorate AKI in LAM. By preserving nitric oxide bioavailability and preventing regional hypoxia, Sildenafil attenuates IRI in canine and swine kidneys [100,186]. Sitaxsentan, an endothelin receptor antagonist, improves hypoxia during AKI in a porcine model [99]. Sodium nitroprusside, a nitric oxide donor, in combination with N-acetyl cysteine and phosphormidon, an endothelin-1 converting enzyme inhibitor, improves renal function after I/R injury in a canine model [188]. Fenoldopam, a synthetic D1 dopamine receptor agonist, demonstrated a prophylactic benefit against the reduction in renal blood flow and renal tubular function during acute hypovolemia in anesthetized dogs [193]. Fenoldopam has also been shown to attenuate I/R-induced AKI in a porcine model [123]. Fenoldopam was found to be beneficial in the prevention or treatment of AKI in postoperative or intensive care patients [200], but a recent review only found that its renoprotective effect is transient [201]. Both atrial natriuretic peptide and brain natriuretic peptide relax vascular smooth muscles and improve blood flow and urine output in dogs [182]. Limited clinical trials suggest that low dose of atrial natriuretic peptide might be effective in preventing or treating AKI [202].

Mesenchymal stem/stromal cell (MSC) therapy has shown promise in ameliorating AKI and stimulating cellular repair in rodents [203]. Emerging evidence has demonstrated similar results in LAM. MSC exhibit recovery and protective function associated with an increase of FoxP3^+^ Treg in cisplatin-induced AKI in *Macaque mulatta* [160]. By reducing renal expression of NF-κB, TNF-α, IFN-γ, MCP-1, and oxidative stress, MSC restores kidney function in atherosclerotic renal artery stenosis-induced AKI in pigs [127]. The main obstacles that must be addressed with MSC are the safety, dose, source of cells, and delivery methods [187,204]. To circumvent some of these obstacles, Lerman and colleagues have shown that intrarenal delivery of MSC extracellular vesicles achieves similar results as MSC in the attenuation of AKI in pigs [128–130]. However, more research is needed.

## Limitations of LAM in AKI studies

Because tools such as genetic modification, monoclonal antibodies, and commercial test kits are not as widely available in LAM as they are in murine models, LAM have been used less for mechanistic inquiries. Further, the high costs of housing, longer breeding cycle, laborious surgical procedures, and animal welfare guidelines discourage the frequent use of LAM. Moreover, the technical expertise needed to surgically manipulate these animals is also challenging. Therefore, LAM is often only utilized for studying AKI for translational purposes. This results in a lack of sufficient knowledge to understand innate and adaptive immune responses behind the results and hinders follow-up studies to refine testing.

While LAM are more similar to each other than they are to rodents, immunology, and physiology still differs among the different species. Therefore, an intervention might not be equally effective in different species of LAM. For example, MSC therapy is reported to be effective in dogs and pigs [187] but not in cats [183] or sheep [179]. Even among the same species, the same treatment can have different results in different AKI models. For example, ATP‐MgCl_2_ was reported to improve I/R-induced AKI [190] but worsen cisplatin-induced nephrotoxicity [191] in dogs. Moreover, in order to induce AKI over a short time frame, the dose of a toxin used in LAM is usually higher than the equivalent dose in humans. The above considerations suggest that a multi-species, staged approach to examine mechanism and efficacy with focused etiological uses would be beneficial.

## Interventions of AKI tested in the porcine models

Use of pigs for biomedical studies was recorded as early as 162 AD when Romans dissected pigs to gain a greater understanding of human physiology [205]. Today, pigs are becoming more and more popular subjects in biomedical research including kidney research (Figure 1). In addition to the immunologic similarities described above, this is due to their out breed nature, proximity to human physical size, and similar anatomy and physiology. Unlike herbivorous rodents, pigs are omnivorous, an advantage to studying the role of gut microbiota in human AKI [95].

Vasculature plays a pivotal role in AKI. Swine kidneys are pyramidal and multilobular with vascular structure comparable to human kidneys, while mice and rats have unilobular kidneys [206]. The anatomy of the swine kidney is actually more similar to humans than that of NHP [207]. The length of renal arteries of large animals is closer to human renal artery length than rodent renal artery length. The average human renal artery is 10.4 cm long, whereas the length of a rat renal artery is only 1.54 cm. In contrast, the average length of the renal artery is 6.1 cm for goats, 3.84 cm for monkeys, and 3.01 cm for pigs [208,209]. Further, pigs have similar renal blood flow rate, resistance index, pulsatility index, and systolic/diastolic index as humans [210]. In the renal superficial veins, humans, cats, and dogs are reported to have ‘stellate veins’, whereas rats have only spur like veins [211]. The porcine renal function analytes such as creatinine, blood urea nitrogen (BUN) and anion secretion [22,23,212] are comparable to human’s. The size of pigs as well as other LAM allow for the serial collection of blood analysis for biomarkers. The National Swine Resource and Research Center (NSRRC) offers triple knockouts and selected transgenic porcine models to facilitate translational research and potential for xenotranplantation [213]. Many markers are available to characterize pig immune cells. Moreover, pigs have been used to troubleshoot isolation of renal progenitor cells, and have been used to examine the effectiveness of these cells [214–216].

While adult domestic pigs are large, difficult to handle, and take a long time to breed, small breeds of pigs offers an alternative that mitigate some of these limitations [206]. In porcine AKI studies, fitting a dose (or ischemic time) with AKI score curve severity is difficult because the sample size is often small. Many therapeutics and their targets that have been tested in swine models to date are summarized in Table 1. Some of the therapeutics listed in the table are preventative, pretreatment, or concomitantly administered, while others are responsive and given as treatment after AKI is established. It is worth mentioning that pigs are traditionally bred for higher fat deposition, which may be protective for AKI [217]. However, in humans obesity is a well-known risk factor for AKI [218].

## Therapies tested in the NHP AKI models

The number of publications utilizing NHP in the kidney research, including AKI, is low (Figure 1). One of the reasons is that due to ethical concerns, testing will only be justified when the treatment or drug has proved effective in other models. The NHP models are often designed for combined clinical insults such as trauma, blood loss, and sepsis, therefore, the injuries are not limited to the kidney. Multiple therapies have shown to improve renal function in NHP AKI models (Table 2). They include cytokine blockade, passive immunotherapy, stem cells, and erythropoietin. Unlike mice [219] and pigs [220,221], miRNA therapies are seldom examined in NHP [222,223]. This is likely because targets and specific gene expression are not well characterized in the renal tissues. Furthermore, kidney targeted drug delivery vehicles are not available [224,225].

## Interventions of AKI tested in ovine models

Publications on the use of sheep models in kidney research, including testing therapies for AKI, are fewer compared to those using pigs but more than publications on the use of cats and dogs combined (Figure 1). One of the reasons is that sheep are an agricultural animal and easier to get approval for studies than cats and dogs. Therapeutics tested in the ovine models are listed in Table 3. The majority of publications are from May and colleagues from Australia.

## Interventions of AKI tested in the feline and canine models

Studies of AKI in cats and dogs are significant in the field of veterinary medicine, but we limited our search to studies relevant for translational purposes. The search results are listed in Table 4. Cats and dogs are not widely used as models of translational AKI research because of ethical guidelines.

## Conclusion

While they are the most commonly used animal models for AKI research, rats and mice have significantly different immune responses and anatomy compared to humans. This heterogeneity is one reason why promising therapeutics developed and tested in these animal models have failed to translate into clinical practice. In contrast, LAM have similarities to humans that may confer advantages when considering potential therapeutics for clinical studies. However, even large animals are not perfect replicas for human AKI. Further, animal experiments are planned in advance and carefully controlled, which is different from the clinical setting where AKI is multifactorial and patients have more variability in terms of comorbidities, age, gender, and genetic diversity [226]. Despite these limitations, the knowledge gleaned from LAM studies has the potential to advance our understanding of the basic pathophysiologic mechanisms of AKI in higher-order animals and to serve as a bridge between murine models and clinical trials.

## Abbreviations

AKI: acute kidney injury
DAMPs: Danger-associated molecular patterns
GFR: Glomerular filtration rate
iNOS: Inducible nitric oxide synthases
I/R: Ischemia reperfusion
IRI: Ischemia reperfusion injury
LAM: Large animal models
LPS: Lipopolysaccharides
MSC: Mesenchymal stem cell
NHP: Nonhuman primates
NO: Nitric oxide
PAMPs: Pathogen-associated molecular patterns
PRR: Pattern recognition receptors
TLR: Toll-like receptors

## References

[1] Stewart IJ, Sosnov JA, Howard JT, et al. Acute kidney injury in critically injured combat veterans: a retrospective cohort study. Am J Kidney Dis. 2016;68(4):564–570. 27155727 10.1053/j.ajkd.2016.03.419

[2] Coca SG, Singanamala S, Parikh CR. Chronic kidney disease after acute kidney injury: a systematic review and meta-analysis. Kidney Int. 2012;81(5):442–448. 22113526 10.1038/ki.2011.379 PMC3788581

[3] Hsu CY, Hsu RK, Yang J, et al. Elevated BP after AKI. JASN. 2016;27(3):914–923. 26134154 10.1681/ASN.2014111114 PMC4769190

[4] Stewart IJ, Sosnov JA, Howard JT, et al. Retrospective analysis of long-term outcomes after combat injury: a hidden cost of war. Circulation. 2015;132(22):2126–2133. 26621637 10.1161/CIRCULATIONAHA.115.016950

[5] Bansal N, Matheny ME, Greevy RA, et al. Acute kidney injury and risk of incident heart failure among US veterans. Am J Kidney Dis. 2018;71(2):236–245. 29162339 10.1053/j.ajkd.2017.08.027

[6] Go AS, Hsu CY, Yang J, et al. Acute kidney injury and risk of heart failure and atherosclerotic events. Clin J Am Soc Nephrol. 2018;13(6):833–841. 29773712 10.2215/CJN.12591117 PMC5989674

[7] Lafrance JP, Miller DR. Acute kidney injury associates with increased long-term mortality. JASN. 2010;21(2):345–352. 20019168 10.1681/ASN.2009060636 PMC2834549

[8] Sawhney S, Marks A, Fluck N, et al. Intermediate and long-term outcomes of survivors of acute kidney injury episodes: a large population-based cohort study. Am J Kidney Dis. 2017;69(1):18–28. 27555107 10.1053/j.ajkd.2016.05.018 PMC5176133

[9] Zarjou A, Sanders PW, Mehta RL, et al. Enabling innovative translational research in acute kidney injury. Clin Transl Sci. 2012;5(1):93–101. 22376265 10.1111/j.1752-8062.2011.00302.x PMC3292183

[10] Di Giorno C, Pinheiro HS, Heinke T, et al. Beneficial effect of N-acetyl-cysteine on renal injury triggered by ischemia and reperfusion. Transplant Proc. 2006;38(9):2774–2776. 17112826 10.1016/j.transproceed.2006.08.178

[11] Campos R, Shimizu MH, Volpini RA, et al. N-acetylcysteine prevents pulmonary edema and acute kidney injury in rats with sepsis submitted to mechanical ventilation. Am J Physiol Lung Cell Mol Physiol. 2012;302(7):L640–50. 22268121 10.1152/ajplung.00097.2011

[12] Kim JH, Lee SS, Jung MH, et al. N-acetylcysteine attenuates glycerol-induced acute kidney injury by regulating MAPKs and Bcl-2 family proteins. Nephrol Dial Transplant. 2010;25(5):1435–1443. 20037173 10.1093/ndt/gfp659

[13] Yenicerioglu Y, Yilmaz O, Sarioglu S, et al. Effects of N-acetylcysteine on radiocontrast nephropathy in rats. Scand J Urol Nephrol. 2006;40(1):63–69. 16452059 10.1080/00365590500329445

[14] Amini M, Salarifar M, Amirbaigloo A, et al. N-acetylcysteine does not prevent contrast-induced nephropathy after cardiac catheterization in patients with diabetes mellitus and chronic kidney disease: a randomized clinical trial. Trials. 2009;10:45. 19563648 10.1186/1745-6215-10-45 PMC2714294

[15] Badri S, Soltani R, Sayadi M, et al. Effect of N-acetylcysteine against vancomycin-induced nephrotoxicity: a randomized controlled clinical trial. Arch Iran Med. 2020;23(6):397–402. 32536177 10.34172/aim.2020.33

[16] Pereira JEG, El Dib R, Braz LG, et al. N-acetylcysteine use among patients undergoing cardiac surgery: a systematic review and meta-analysis of randomized trials. PLoS One. 2019;14(5):e0213862. 31071081 10.1371/journal.pone.0213862 PMC6508704

[17] Adabag AS, Ishani A, Koneswaran S, et al. Utility of N-acetylcysteine to prevent acute kidney injury after cardiac surgery: a randomized controlled trial. Am Heart J. 2008;155(6):1143–1149. 18513531 10.1016/j.ahj.2008.01.013

[18] Mei M, Zhao HW, Pan QG, Pu YM, et al. Efficacy of N-acetylcysteine in preventing acute kidney injury after cardiac surgery: a meta-analysis study. J Invest Surg. 2018;31(1):14–23. 28060555 10.1080/08941939.2016.1269853

[19] Skrypnyk NI, Siskind LJ, Faubel S, et al. Bridging translation for acute kidney injury with better preclinical modeling of human disease. Am J Physiol Renal Physiol. 2016;310(10):F972–84. 26962107 10.1152/ajprenal.00552.2015 PMC4889323

[20] de Caestecker M, Humphreys BD, Liu KD, et al.; ASN AKI Advisory Group. Bridging translation by improving preclinical study design in AKI. J Am Soc Nephrol. 2015;26(12):2905–2916. 26538634 10.1681/ASN.2015070832 PMC4657852

[21] Mestas J, Hughes CC. Of mice and not men: differences between mouse and human immunology. J Immunol. 2004;172(5):2731–2738. 14978070 10.4049/jimmunol.172.5.2731

[22] Meurens F, Summerfield A, Nauwynck H, et al. The pig: a model for human infectious diseases. Trends Microbiol. 2012;20(1):50–57. 22153753 10.1016/j.tim.2011.11.002 PMC7173122

[23] Dawson HD, Smith AD, Chen C, et al. An in-depth comparison of the porcine, murine and human inflammasomes; lessons from the porcine genome and transcriptome. Vet Microbiol. 2017;202:2–15. 27321134 10.1016/j.vetmic.2016.05.013

[24] Smirnova I, Poltorak A, Chan EK, et al. Phylogenetic variation and polymorphism at the toll-like receptor 4 locus (TLR4). Genome Biol. 2000;1(1):RESEARCH002. 11104518 10.1186/gb-2000-1-1-research002 PMC31919

[25] Barreiro LB, Marioni JC, Blekhman R, et al. Functional comparison of innate immune signaling pathways in primates. PLoS Genet. 2010;6(12):e1001249. 21187902 10.1371/journal.pgen.1001249 PMC3002988

[26] Star RA. Treatment of acute renal failure. Kidney Int. 1998;54(6):1817–1831. 9853246 10.1046/j.1523-1755.1998.00210.x

[27] Chopra TA, Brooks CH, Okusa MD. Acute kidney injury prevention. Contrib Nephrol. 2016;187:9–23. 26881939 10.1159/000443152

[28] Zuk A, Bonventre JV. Acute kidney injury. Annu Rev Med. 2016;67:293–307. 26768243 10.1146/annurev-med-050214-013407 PMC4845743

[29] Burmeister DM, Gomez BI, Dubick MA. Molecular mechanisms of trauma-induced acute kidney injury: Inflammatory and metabolic insights from animal models. Biochim Biophys Acta Mol Basis Dis. 2017;1863(10 Pt B):2661–2671. 28431991 10.1016/j.bbadis.2017.04.011

[30] Yang Y, Song M, Liu Y, et al. Renoprotective approaches and strategies in acute kidney injury. Pharmacol Ther. 2016;163:58–73. 27108948 10.1016/j.pharmthera.2016.03.015 PMC5123830

[31] Agarwal A, Dong Z, Harris R, et al.; Acute Dialysis Quality Initiative XIII Working Group. Cellular and molecular mechanisms of aKI. J Am Soc Nephrol. 2016;27(5):1288–1299. 26860342 10.1681/ASN.2015070740 PMC4849836

[32] Rosin DL, Okusa MD. Dangers within: DAMP responses to damage and cell death in kidney disease. J Am Soc Nephrol. 2011;22(3):416–425. 21335516 10.1681/ASN.2010040430 PMC4493973

[33] Ishimoto Y, Inagi R. Mitochondria: a therapeutic target in acute kidney injury. Nephrol Dial Transplant. 2016;31(7):1062–1069. 26333547 10.1093/ndt/gfv317

[34] Li L, Okusa MD. Macrophages, dendritic cells, and kidney ischemia-reperfusion injury. Semin Nephrol. 2010;30(3):268–277. 20620671 10.1016/j.semnephrol.2010.03.005 PMC2904394

[35] Jang HR, Rabb H. Immune cells in experimental acute kidney injury. Nat Rev Nephrol. 2015;11(2):88–101. 25331787 10.1038/nrneph.2014.180

[36] Wohlfahrtova M, Tycova I, Honsova E, et al. Molecular patterns of subclinical and clinical rejection of kidney allograft: quantity matters. Kidney Blood Press Res. 2015;40(3):244–257. 25997515 10.1159/000368500

[37] Lever JM, Hull TD, Boddu R, et al. Resident macrophages reprogram toward a developmental state after acute kidney injury. JCI Insight. 2019;4(2):e125503. 10.1172/jci.insight.125503 PMC6413788 30674729

[38] Prasada R, Muktesh G, Samanta J, et al. Natural history and profile of selective cytokines in patients of acute pancreatitis with acute kidney injury. Cytokine. 2020;133:155177. 32593952 10.1016/j.cyto.2020.155177

[39] de Fontnouvelle CA, Greenberg JH, Thiessen-Philbrook HR, et al. Interleukin-8 and tumor necrosis factor predict acute kidney injury after pediatric cardiac surgery. Ann Thorac Surg. 2017;104(6):2072–2079. 28821332 10.1016/j.athoracsur.2017.04.038 PMC5696070

[40] Sirota JC, Walcher A, Faubel S, et al. Urine IL-18, NGAL, IL-8 and serum IL-8 are biomarkers of acute kidney injury following liver transplantation. BMC Nephrol. 2013;14:17. 23327592 10.1186/1471-2369-14-17 PMC3562144

[41] Iwakura T, Zhao Z, Marschner JA, et al. Dipeptidyl peptidase-4 inhibitor teneligliptin accelerates recovery from cisplatin-induced acute kidney injury by attenuating inflammation and promoting tubular regeneration. Nephrol Dial Transplant. 2019;34(10):1669–1680. 30624740 10.1093/ndt/gfy397

[42] Atri C, Guerfali FZ, Laouini D. Role of human macrophage polarization in inflammation during infectious diseases. IJMS. 2018;19(6):1801. 10.3390/ijms19061801 PMC6032107 29921749

[43] Huen SC, Cantley LG. Macrophages in renal injury and repair. Annu Rev Physiol. 2017;79:449–469. 28192060 10.1146/annurev-physiol-022516-034219

[44] Zschaler J, Schlorke D, Arnhold J. Differences in innate immune response between man and mouse. Crit Rev Immunol. 2014;34(5):433–454. 25404048

[45] Roncal CA, Mu W, Croker B, et al. Effect of elevated serum uric acid on cisplatin-induced acute renal failure. Am J Physiol Renal Physiol. 2007;292(1):F116–22. 17210794 10.1152/ajprenal.00160.2006

[46] Ejaz AA, Dass B, Lingegowda V, et al. Effect of uric acid lowering therapy on the prevention of acute kidney injury in cardiovascular surgery. Int Urol Nephrol. 2013;45(2):449–458. 22648289 10.1007/s11255-012-0192-2

[47] Ding C, Han F, Xiang H, et al. Probiotics ameliorate renal ischemia-reperfusion injury by modulating the phenotype of macrophages through the IL-10/GSK-3β/PTEN signaling pathway. Pflugers Arch. 2019;471(4):573–581. 30426249 10.1007/s00424-018-2213-1

[48] Kim MG, Lim K, Lee YJ, et al. M2 macrophages predict worse long-term outcomes in human acute tubular necrosis. Sci Rep. 2020;10(1):2122. 32034190 10.1038/s41598-020-58725-w PMC7005727

[49] Palmer MB, Vichot AA, Cantley LG, et al. Quantification and localization of M2 macrophages in human kidneys with acute tubular injury. Int J Nephrol Renovasc Dis. 2014;7:415–419. 25404860 10.2147/IJNRD.S66936 PMC4230184

[50] Hu W, Lin J, Lian X, et al. M2a and M2b macrophages predominate in kidney tissues and M2 subpopulations were associated with the severity of disease of IgAN patients. Clin Immunol. 2019;205:8–15. 31078708 10.1016/j.clim.2019.05.005

[51] Costa JS, Alves R, Sousa V, et al. Fibrogenesis in kidney transplant: dysfunction progress biomarkers. Transplant Proc. 2017;49(4):787–791. 28457395 10.1016/j.transproceed.2017.01.063

[52] Li J, Liu CH, Xu DL, et al. Significance of CD163-positive macrophages in proliferative glomerulonephritis. Am J Med Sci. 2015;350(5):387–392. 26379042 10.1097/MAJ.0000000000000569

[53] Wang S, Zhang C, Li J, et al. Erythropoietin protects against rhabdomyolysis-induced acute kidney injury by modulating macrophage polarization. Cell Death Dis. 2017;8(4):e2725. 28383559 10.1038/cddis.2017.104 PMC5477572

[54] Russell PS, Hong J, Windsor JA, et al. Renal lymphatics: anatomy, physiology, and clinical implications. Front Physiol. 2019;10:251. 30923503 10.3389/fphys.2019.00251 PMC6426795

[55] Pabst R. The pig as a model for immunology research. Cell Tissue Res. 2020;380(2):287–304. 32356014 10.1007/s00441-020-03206-9 PMC7223737

[56] Fairbairn L, Kapetanovic R, Sester DP, et al. The mononuclear phagocyte system of the pig as a model for understanding human innate immunity and disease. J Leukoc Biol. 2011;89(6):855–871. 21233410 10.1189/jlb.1110607

[57] Gómez BI, Harrington BK, Chao T, et al. Impact of oral resuscitation on circulating and splenic leukocytes after burns. Burns. 2020;46(3):567–578. 31787475 10.1016/j.burns.2019.08.019

[58] Reynolds PS, Fisher BJ, McCarter J, et al. Interventional vitamin C: a strategy for attenuation of coagulopathy and inflammation in a swine multiple injuries model. J Trauma Acute Care Surg. 2018;85(1S Suppl 2):S57–S67. 29538225 10.1097/TA.0000000000001844

[59] Castellano G, Melchiorre R, Loverre A, et al. Therapeutic targeting of classical and lectin pathways of complement protects from ischemia-reperfusion-induced renal damage. Am J Pathol. 2010;176(4):1648–1659. 20150432 10.2353/ajpath.2010.090276 PMC2843457

[60] Thomas AV, Broers AD, Vandegaart HF, et al. Genomic structure, promoter analysis and expression of the porcine (*Sus scrofa*) TLR4 gene. Mol Immunol. 2006;43(6):653–659. 15869793 10.1016/j.molimm.2005.04.001

[61] Vaure C, Liu Y. A comparative review of toll-like receptor 4 expression and functionality in different animal species. Front Immunol. 2014;5:316. 25071777 10.3389/fimmu.2014.00316 PMC4090903

[62] Tohno M, Shimazu T, Aso H, et al. Molecular cloning and functional characterization of porcine MyD88 essential for TLR signaling. Cell Mol Immunol. 2007;4(5):369–376. 17976317

[63] CSaA C. Initial sequence of the chimpanzee genome and comparison with the human genome. Nature. 2005;437(7055):69–87. 16136131 10.1038/nature04072

[64] Gharaie Fathabad S, Kurzhagen JT, Sadasivam M, et al. T lymphocytes in acute kidney injury and repair. Semin Nephrol. 2020;40(2):114–125. 32303275 10.1016/j.semnephrol.2020.01.003

[65] Delpech PO, Thuillier R, SaintYves T, et al. Inhibition of complement improves graft outcome in a pig model of kidney autotransplantation. J Transl Med. 2016;14(1):277. 27663514 10.1186/s12967-016-1013-7 PMC5035455

[66] Faure JP, Petit I, Zhang K, et al. Protective roles of polyethylene glycol and trimetazidine against cold ischemia and reperfusion injuries of pig kidney graft. Am J Transplant. 2004;4(4):495–504. 15023141 10.1111/j.1600-6143.2004.00365.x

[67] Faure JP, Baumert H, Han Z, et al. Evidence for a protective role of trimetazidine during cold ischemia: targeting inflammation and nephron mass. Biochem Pharmacol. 2003;66(11):2241–2250. 14609748 10.1016/j.bcp.2003.07.011

[68] Hauet T, Goujon JM, Baumert H, et al. Polyethylene glycol reduces the inflammatory injury due to cold ischemia/reperfusion in autotransplanted pig kidneys. Kidney Int. 2002;62(2):654–667. 12110031 10.1046/j.1523-1755.2002.00473.x

[69] Kinsey GR, Huang L, Vergis AL, et al. Regulatory T cells contribute to the protective effect of ischemic preconditioning in the kidney. Kidney Int. 2010;77(9):771–780. 20164824 10.1038/ki.2010.12 PMC2912287

[70] Lai LW, Yong KC, Lien YH. Pharmacologic recruitment of regulatory T cells as a therapy for ischemic acute kidney injury. Kidney Int. 2012;81(10):983–992. 22189844 10.1038/ki.2011.412 PMC3340526

[71] De Greef KE, Ysebaert DK, Dauwe S, et al. Anti-B7-1 blocks mononuclear cell adherence in vasa recta after ischemia. Kidney Int. 2001;60(4):1415–1427. 11576355 10.1046/j.1523-1755.2001.00944.x

[72] Ysebaert DK, De Greef KE, De Beuf A, et al. T cells as mediators in renal ischemia/reperfusion injury. Kidney Int. 2004;66(2):491–496. 15253695 10.1111/j.1523-1755.2004.761_4.x

[73] Sayegh MH, Akalin E, Hancock WW, et al. CD28-B7 blockade after alloantigenic challenge in vivo inhibits Th1 cytokines but spares Th2. J Exp Med. 1995;181(5):1869–1874. 7536798 10.1084/jem.181.5.1869 PMC2192009

[74] Dekel B, Böcher WO, Marcus H, et al. Acute cellular rejection of human renal tissue by adoptive transfer of allogeneic human peripheral blood mononuclear cells into chimeric rats: sequential gene expression of cytokines, chemokines and cytolytic effector molecules, and their regulation by CTLA-4-Ig. Int Immunol. 1999;11(10):1673–1683. 10508185 10.1093/intimm/11.10.1673

[75] Magee DE, Hird AE, Klaassen Z, et al. Adverse event profile for immunotherapy agents compared with chemotherapy in solid organ tumors: a systematic review and meta-analysis of randomized clinical trials. Ann Oncol. 2020;31(1):50–60. 31912796 10.1016/j.annonc.2019.10.008

[76] Meraz-Muñoz A, Amir E, Ng P, et al. Acute kidney injury associated with immune checkpoint inhibitor therapy: incidence, risk factors and outcomes. J Immunother Cancer. 2020;8(1):e000467. 32601079 10.1136/jitc-2019-000467 PMC7326260

[77] Dellepiane S, Leventhal JS, Cravedi P. T cells and acute kidney injury: a two-way relationship. Front Immunol. 2020;11:1546. 32765535 10.3389/fimmu.2020.01546 PMC7379378

[78] Paulissen SM, van Hamburg JP, Dankers W, et al. The role and modulation of CCR6+ Th17 cell populations in rheumatoid arthritis. Cytokine. 2015;74(1):43–53. 25828206 10.1016/j.cyto.2015.02.002

[79] Maeda S, Osaga S, Maeda T, et al. Circulating Th17.1 cells as candidate for the prediction of therapeutic response to abatacept in patients with rheumatoid arthritis: an exploratory research. PLoS One. 2019;14(11):e0215192. 31747403 10.1371/journal.pone.0215192 PMC6867595

[80] Sachs DH. Tolerance: of mice and men. J Clin Invest. 2003;111(12):1819–1821. 12813017 10.1172/JCI18926 PMC161433

[81] Haanstra KG, van der Maas MJ, T Hart BA, et al. Characterization of naturally occurring CD4 + CD25+ regulatory T cells in rhesus monkeys. Transplantation. 2008;85(8):1185–1192. 18431240 10.1097/TP.0b013e31816b15b9

[82] Burr AHP, Bhattacharjee A, Hand TW. Nutritional modulation of the microbiome and immune response. J Immunol. 2020;205(6):1479–1487. 32900885 10.4049/jimmunol.2000419 PMC7490768

[83] Yang Q, Wang Y, Jia A, et al. The crosstalk between gut bacteria and host immunity in intestinal inflammation. J Cell Physiol. 2020. doi:10.1002/jcp.30024 32853458

[84] Knauf F, Brewer JR, Flavell RA. Immunity, microbiota and kidney disease. Nat Rev Nephrol. 2019;15(5):263–274. 30796361 10.1038/s41581-019-0118-7

[85] Gharaie S, Noel S, Rabb H. Gut microbiome and AKI: roles of the immune system and short-chain fatty acids. Nephron. 2020:1–3. doi:10.1159/000508984 32721962

[86] Gong J, Noel S, Pluznick JL, et al. Gut microbiota-kidney cross-talk in acute kidney injury. Semin Nephrol. 2019;39(1):107–116. 30606403 10.1016/j.semnephrol.2018.10.009 PMC6322425

[87] Zhang J, Ankawi G, Sun J, et al. Gut-kidney crosstalk in septic acute kidney injury. Crit Care. 2018;22(1):117. 29724256 10.1186/s13054-018-2040-y PMC5934860

[88] Andrianova NV, Popkov VA, Klimenko NS, et al. Microbiome-metabolome signature of acute kidney injury. Metabolites. 2020;10(4):142. 10.3390/metabo10040142 PMC7241241 32260384

[89] Emal D, Rampanelli E, Stroo I, et al. Depletion of gut microbiota protects against renal ischemia-reperfusion injury. J Am Soc Nephrol. 2017;28(5):1450–1461. 27927779 10.1681/ASN.2016030255 PMC5407717

[90] Nakade Y, Iwata Y, Furuichi K, et al. Gut microbiota-derived D-serine protects against acute kidney injury. JCI Insight. 2018;3(20):e97957. 10.1172/jci.insight.97957 PMC6237464 30333299

[91] Yang J, Kim CJ, Go YS, et al. Intestinal microbiota control acute kidney injury severity by immune modulation. Kidney Int. 2020;S0085–2538(20)30553–30556. 10.1016/j.kint.2020.04.048 32470493

[92] Nagpal R, Wang S, Solberg Woods LC, et al. Comparative microbiome signatures and short-chain fatty acids in mouse, rat, non-human primate, and human feces. Front Microbiol. 2018;9:2897. 30555441 10.3389/fmicb.2018.02897 PMC6283898

[93] Hugenholtz F, de Vos WM. Mouse models for human intestinal microbiota research: a critical evaluation. Cell Mol Life Sci. 2018;75(1):149–160. 29124307 10.1007/s00018-017-2693-8 PMC5752736

[94] Li X, Liang S, Xia Z, et al. Establishment of a Macaca fascicularis gut microbiome gene catalog and comparison with the human, pig, and mouse gut microbiomes. Gigascience. 2018;7(9):giy100. 10.1093/gigascience/giy100 PMC6137240 30137359

[95] Heinritz SN, Mosenthin R, Weiss E. Use of pigs as a potential model for research into dietary modulation of the human gut microbiota. Nutr Res Rev. 2013;26(2):191–209. 24134811 10.1017/S0954422413000152

[96] Wang M, Donovan SM. Human microbiota-associated swine: current progress and future opportunities. Ilar J. 2015;56(1):63–73. 25991699 10.1093/ilar/ilv006 PMC7108572

[97] Labossiere JR, Pelletier J-S, Thiesen A, et al. Doxycycline attenuates renal injury in a swine model of neonatal hypoxia-reoxygenation. Shock. 2015;43(1):99–105. 25105465 10.1097/SHK.0000000000000257

[98] Goebel U, Siepe M, Schwer CI, et al. Inhaled carbon monoxide prevents acute kidney injury in pigs after cardiopulmonary bypass by inducing a heat shock response. Anesth Analgesia. 2010;111(1):29–37. 10.1213/ANE.0b013e3181e0cca4 20519418

[99] Patel NN, Toth T, Jones C, et al. Prevention of post-cardiopulmonary bypass acute kidney injury by endothelin A receptor blockade. Critic Care Med. 2011;39(4):793–802. 10.1097/CCM.0b013e318206d563 21220998

[100] Patel NN, Lin H, Toth T, et al. Phosphodiesterase-5 inhibition prevents postcardiopulmonary bypass acute kidney injury in swine. Ann Thoracic Surg. 2011;92(6):2168–2176. 10.1016/j.athoracsur.2011.07.002 21983073

[101] Wang X, Xue Q, Yan F, et al. Ulinastatin protects against acute kidney injury in infant piglets model undergoing surgery on hypothermic low-flow cardiopulmonary bypass. PloS One. 2015;10(12):e0144516. 26656098 10.1371/journal.pone.0144516 PMC4684368

[102] Liu S, Xu J, Gao Y, et al. Multi-organ protection of ulinastatin in traumatic cardiac arrest model. World J Emerg Surg. 2018;13:51. 30459824 10.1186/s13017-018-0212-3 PMC6233498

[103] Davidson JA, Khailova L, Treece A, et al. Alkaline phosphatase treatment of acute kidney injury in an infant piglet model of cardiopulmonary bypass with deep hypothermic circulatory arrest. Sci Rep. 2019;9(1):1–14. 31578351 10.1038/s41598-019-50481-w PMC6775126

[104] Barrera-Chimal J, André-Grégoire G, Nguyen Dinh Cat A, et al. Benefit of mineralocorticoid receptor antagonism in AKI: role of vascular smooth muscle Rac1. J Am Soc Nephrol. 2017;28(4):1216–1226. 28087726 10.1681/ASN.2016040477 PMC5373452

[105] Jayle C, Milinkevitch S, Favreau F, et al. Protective role of selectin ligand inhibition in a large animal model of kidney ischemia-reperfusion injury. Kidney Int. 2006;69(10):1749–1755. 16625150 10.1038/sj.ki.5000335

[106] Sølling C, Christensen AT, Krag S, et al. Erythropoietin administration is associated with short-term improvement in glomerular filtration rate after ischemia-reperfusion injury. Acta Anaesthesiol Scand. 2011;55(2):185–195. 21226860 10.1111/j.1399-6576.2010.02369.x

[107] Simon F, Scheuerle A, Calzia E, et al. Erythropoietin during porcine aortic balloon occlusion-induced ischemia/reperfusion injury. Crit Care Med. 2008;36(7):2143–2150. 18552697 10.1097/CCM.0b013e31817d7912

[108] Matějková Š, Scheuerle A, Wagner F, et al. Carbamylated erythropoietin-FC fusion protein and recombinant human erythropoietin during porcine kidney ischemia/reperfusion injury. Intens Care Med. 2013;39(3):497–510. 10.1007/s00134-012-2766-y 23291730

[109] van Rijt WG, Nieuwenhuijs-Moeke GJ, van Goor H, et al. ARA290, a non-erythropoietic EPO derivative, attenuates renal ischemia/reperfusion injury. J Transl Med. 2013;11(1):9. 23302512 10.1186/1479-5876-11-9 PMC3567997

[110] Yang C, Hosgood SA, Meeta P, et al. Cyclic helix B peptide in preservation solution and autologous blood perfusate ameliorates ischemia-reperfusion injury in isolated porcine kidneys. Transplant Direct. 2015;1(2):e6. 27500213 10.1097/TXD.0000000000000515 PMC4946457

[111] Simmons MN, Subramanian V, Crouzet S, et al. Alpha-melanocyte stimulating hormone analogue AP214 protects against ischemia induced acute kidney injury in a porcine surgical model . J Urol. 2010;183(4):1625–1629. 20172543 10.1016/j.juro.2009.12.007

[112] Cau J, Favreau F, Zhang K, et al. FR167653 improves renal recovery and decreases inflammation and fibrosis after renal ischemia reperfusion injury. J Vasc Surg. 2009;49(3):728–740. 19268775 10.1016/j.jvs.2008.09.056

[113] Jayle C, Faure JP, Thuillier R, et al. Influence of nephron mass and a phosphorylated 38 mitogen-activated protein kinase inhibitor on the development of early and long-term injury after renal warm ischaemia. Br J Surg. 2009;96(7):799–808. 19526623 10.1002/bjs.6589

[114] Doucet C, Milin S, Favreau F, et al. A p38 mitogen-activated protein kinase inhibitor protects against renal damage in a non-heart-beating donor model. Am J Physiol Renal Physiol. 2008;295(1):F179–91. 18448593 10.1152/ajprenal.00252.2007

[115] Miura K, Sahara H, Sekijima M, et al. Protective effect of neutralization of the extracellular high-mobility group box 1 on renal ischemia-reperfusion injury in miniature swine. Transplantation. 2014;98(9):937–943. 25136847 10.1097/TP.0000000000000358

[116] Yang C, Zhao T, Zhao Z, et al. Serum-stabilized naked caspase-3 siRNA protects autotransplant kidneys in a porcine model. Mol Ther. 2014;22(10):1817–1828. 24930602 10.1038/mt.2014.111 PMC4428396

[117] Yang B, Hosgood SA, Harper SJ, et al. Leucocyte depletion improves renal function in porcine kidney hemoreperfusion through reduction of myeloperoxidase + cells, caspase-3, IL-1β, and tubular apoptosis. J Surg Res. 2010;164(2):e315–e324. 20869081 10.1016/j.jss.2010.07.044

[118] Simon F, Scheuerle A, Gröger M, et al. Effects of intravenous sulfide during porcine aortic occlusion-induced kidney ischemia/reperfusion injury. Shock. 2011;35(2):156–163. 20661185 10.1097/SHK.0b013e3181f0dc91

[119] Satterly SA, Salgar S, Hoffer Z, et al. Hydrogen sulfide improves resuscitation via non-hibernatory mechanisms in a porcine shock model. J Surg Res. 2015;199(1):197–210. 25956183 10.1016/j.jss.2015.04.001

[120] Hosgood SA, Moore T, Qurashi M, et al. Hydrogen gas does not ameliorate renal ischemia reperfusion injury in a preclinical model. Artif Organs. 2018;42(7):723–727. 29611214 10.1111/aor.13118

[121] Xu M, Wang X, Banan B, et al. Anti-CD47 monoclonal antibody therapy reduces ischemia-reperfusion injury of renal allografts in a porcine model of donation after cardiac death. Am J Transplant. 2018;18(4):855–867. 29087049 10.1111/ajt.14567 PMC5878700

[122] Kolsrud O, Damén T, Nygren A, et al. Effects of atrial natriuretic peptide on renal function during cardiopulmonary bypass: a randomized pig model. Eur J Cardiothorac Surg. 2020;57(4):652–659. 31711139 10.1093/ejcts/ezz297

[123] Miller Q, Peyton BD, Cohn EJ, et al. The effects of intraoperative fenoldopam on renal blood flow and tubular function following suprarenal aortic cross-clamping. Ann Vasc Surg. 2003;17(6):656–662. 14569432 10.1007/s10016-003-0067-1

[124] Gozdzik W, Zielinski S, Zielinska M, et al. Beneficial effects of inhaled nitric oxide with intravenous steroid in an ischemia-reperfusion model involving aortic clamping. Int J Immunopathol Pharmacol. 2018;32:394632017751486. 29376749 10.1177/0394632017751486 PMC5851102

[125] Cau J, Favreau F, Tillement JP, et al. Trimetazidine reduces early and long-term effects of experimental renal warm ischemia: a dose effect study. J Vasc Surg. 2008;47(4):852–860. 18280092 10.1016/j.jvs.2007.10.036

[126] Kim M-J, Lee S-J, Park C-S, et al. Attenuation of renal ischemia-reperfusion injury by antioxidant vitamins in pigs. J Vet Clin. 2007;24(2):94–98.

[127] Eirin A, Zhu XY, Krier JD, et al. Adipose tissue-derived mesenchymal stem cells improve revascularization outcomes to restore renal function in swine atherosclerotic renal artery stenosis. Stem Cells. 2012;30(5):1030–1041. 22290832 10.1002/stem.1047 PMC3694782

[128] Aghajani Nargesi A, Lerman LO, Eirin A. Mesenchymal stem cell-derived extracellular vesicles for kidney repair: current status and looming challenges. Stem Cell Res Ther. 2017;8(1):273. 29202871 10.1186/s13287-017-0727-7 PMC5713024

[129] Eirin A, Zhu XY, Puranik AS, et al. Mesenchymal stem cell-derived extracellular vesicles attenuate kidney inflammation. Kidney Int. 2017;92(1):114–124. 28242034 10.1016/j.kint.2016.12.023 PMC5483390

[130] Zhao Y, Zhu X, Zhang L, et al. Mesenchymal stem/stromal cells and their extracellular vesicle progeny decrease injury in post-stenotic swine kidney through different mechanisms. Stem Cells Dev. 2020;29(18):1190–1200. 32657229 10.1089/scd.2020.0030 PMC7482134

[131] Doulamis IP, Guariento A, Duignan T, et al. Mitochondrial transplantation by intra-arterial injection for acute kidney injury. Am J Physiol Renal Physiol. 2020;319(3):F403–F413. 32686525 10.1152/ajprenal.00255.2020 PMC7509287

[132] Kishi S, Campanholle G, Gohil VM, et al. Meclizine preconditioning protects the kidney against ischemia-reperfusion injury. EBioMedicine. 2015;2(9):1090–1101. 26501107 10.1016/j.ebiom.2015.07.035 PMC4588407

[133] Johnson ST, Bigam DL, Emara M, et al. N-acetylcysteine improves the hemodynamics and oxidative stress in hypoxic newborn pigs reoxygenated with 100% oxygen. Shock. 2007;28(4):484–490. 17577140 10.1097/shk.0b013e31804f775d

[134] Lee TF, Liu JQ, Li YQ, et al. Improved renal recovery with postresuscitation N-acetylcysteine treatment in asphyxiated newborn pigs. Shock. 2011;35(4):428–433. 20938377 10.1097/SHK.0b013e3181fffec2

[135] Kuntscher V, Treska V, Racek J, et al. Does the administration of antioxidants as scavengers of reactive oxygen species in kidney transplantation really have sense? Bratisl Lek Listy. 2007;108(9):385–387. 18225474

[136] Soussi D, Danion J, Baulier E, et al. Vectisol formulation enhances solubility of resveratrol and brings its benefits to kidney transplantation in a preclinical porcine model. IJMS. 2019;20(9):2268. 10.3390/ijms20092268 PMC6540035 31071925

[137] Kim SR, Erin A, Zhang X, et al. Mitochondrial protection partly mitigates kidney cellular senescence in swine atherosclerotic renal artery stenosis. Cell Physiol Biochem. 2019;52(3):617. 30907989 10.33594/000000044 PMC6519989

[138] Amdisen C, Keller AK, Hansen RS, et al. Testing danegaptide effects on kidney function after ischemia/reperfusion injury in a new porcine two week model. PLoS One. 2016;11(10):e0164109. 27760220 10.1371/journal.pone.0164109 PMC5070773

[139] Soni H, Peixoto-Neves D, Olushoga MA, et al. Pharmacological inhibition of TRPV4 channels protects against ischemia-reperfusion-induced renal insufficiency in neonatal pigs. Clin Sci (Lond). 2019;133(9):CS20180815. 30988131 10.1042/CS20180815 PMC11250923

[140] Cui J, Bai X-Y, Sun X, et al. Rapamycin protects against gentamicin-induced acute kidney injury via autophagy in mini-pig models. Sci Rep. 2015;5:11256. 26052900 10.1038/srep11256 PMC4459224

[141] Kumar G, Solanki MH, Xue X, et al. Magnesium improves cisplatin-mediated tumor killing while protecting against cisplatin-induced nephrotoxicity. Am J Physiol Renal Physiol. 2017;313(2):F339–f350. 28424213 10.1152/ajprenal.00688.2016

[142] Wu J, Wan X, Zhang H, et al. Retinoic acid attenuates contrast-induced acute kidney injury in a miniature pig model. Biochem Biophys Res Commun. 2019;512(2):163–169. 30878186 10.1016/j.bbrc.2019.03.013

[143] Xu J, Ma L, Fu P. MicroRNA-30c attenuates contrast-induced acute kidney injury by suppressing NLRP3 inflammasome. Int Immunopharmacol. 2020;87:106457. 32682254 10.1016/j.intimp.2020.106457

[144] Cui J, Tang L, Hong Q, et al. N-acetylcysteine ameliorates gentamicin-induced nephrotoxicity by enhancing autophagy and reducing oxidative damage in miniature pigs. Shock. 2019;52(6):622–630. 30676497 10.1097/SHK.0000000000001319 PMC6855429

[145] Junot S, Keroak S, Del Castillo JR, et al. Inhaled nitric oxide prevents NSAID-induced renal impairment in pseudo-normovolaemic piglets. PloS One. 2017;12(6):e0179475. 28658254 10.1371/journal.pone.0179475 PMC5489163

[146] Wagner KE, Martinez JM, Vath SD, et al. Early immunoneutralization of calcitonin precursors attenuates the adverse physiologic response to sepsis in pigs. Crit Care Med. 2002;30(10):2313–2321. 12394961 10.1097/00003246-200210000-00021

[147] Sølling C, Christensen AT, Nygaard U, et al. Erythropoietin does not attenuate renal dysfunction or inflammation in a porcine model of endotoxemia. Acta Anaesthesiol Scand. 2011;55(4):411–421. 21342148 10.1111/j.1399-6576.2011.02396.x

[148] Yeh YC, Yu LC, Wu CY, et al.; NTUH Center of Microcirculation Medical Research (NCMMR). Effects of endotoxin absorber hemoperfusion on microcirculation in septic pigs. J Surg Res. 2017;211:242–250. 28501124 10.1016/j.jss.2016.12.026

[149] Kubiak BD, Albert SP, Gatto LA, et al. Peritoneal negative pressure therapy prevents multiple organ injury in a chronic porcine sepsis and ischemia/reperfusion model. Shock. 2010;34(5):525–534. 20823698 10.1097/SHK.0b013e3181e14cd2

[150] Gomez BI, McIntyre MK, Gurney JM, et al. Enteral resuscitation with oral rehydration solution to reduce acute kidney injury in burn victims: evidence from a porcine model. PLoS One. 2018;13(5):e0195615. 29718928 10.1371/journal.pone.0195615 PMC5931460

[151] Smith S, Behrens B, McCully B, et al. Aggressive treatment of acute kidney injury and hyperkalemia improves survival in a combat relevant trauma model in swine. Am J Surg. 2020;219(5):860–864. 32245610 10.1016/j.amjsurg.2020.02.058

[152] de Castro LUC, Ida KK, Otsuki DA, et al. Vasopressin analog terlipressin attenuates kidney injury in hemorrhagic shock. Trauma Surg Acute Care Open. 2016;1(1):e000039. 29766070 10.1136/tsaco-2016-000039 PMC5891712

[153] van Griensven M, Ricklin D, Denk S, et al. Protective effects of the complement inhibitor compstatin CP40 in hemorrhagic shock. Shock. 2019;51(1):78–87. 29461464 10.1097/SHK.0000000000001127 PMC6092248

[154] Halbgebauer R, Karasu E, Braun CK, et al. Thirty-eight-negative kinase 1 is a mediator of acute kidney injury in experimental and clinical traumatic hemorrhagic shock. Front Immunol. 2020;11:2081. 32983160 10.3389/fimmu.2020.02081 PMC7479097

[155] Feng L, He G, Cai L, et al. Artificial liver and renal support system for cynomolgus monkeys with surgery-induced acute renal failure: a preclinical study. Biomed Res Int. 2018;2018:7456898. 29992160 10.1155/2018/7456898 PMC5994316

[156] Ishii Y, Sawada T, Murakami T, et al. Renoprotective effect of erythropoietin against ischaemia-reperfusion injury in a non-human primate model. Nephrol Dial Transplant. 2011;26(4):1157–1162. 20935018 10.1093/ndt/gfq601

[157] Qi S, Xu D, Ma A, et al. Effect of a novel inducible nitric oxide synthase inhibitor, FR260330, in prevention of renal ischemia/reperfusion injury in vervet monkeys. Transplantation. 2006;81(4):627–631. 16495814 10.1097/01.tp.0000199282.05021.0c

[158] Dehnadi A, Benedict Cosimi A, Neal Smith R, et al. Prophylactic orthosteric inhibition of leukocyte integrin CD11b/CD18 prevents long-term fibrotic kidney failure in cynomolgus monkeys. Nat Commun. 2017;8:13899. 28071653 10.1038/ncomms13899 PMC5234083

[159] Lee KW, Kim TM, Kim KS, et al. Renal ischemia-reperfusion injury in a diabetic monkey model and therapeutic testing of human bone marrow-derived mesenchymal stem cells. J Diabetes Res. 2018;2018:5182606. 30155487 10.1155/2018/5182606 PMC6092988

[160] Moghadasali R, Azarnia M, Hajinasrollah M, et al. Intra-renal arterial injection of autologous bone marrow mesenchymal stromal cells ameliorates cisplatin-induced acute kidney injury in a rhesus Macaque mulatta monkey model. Cytotherapy. 2014;16(6):734–749. 24801377 10.1016/j.jcyt.2014.01.004

[161] Gautier J-C, Zhou X, Yang Y, et al. Evaluation of novel biomarkers of nephrotoxicity in Cynomolgus monkeys treated with gentamicin. Toxicol Appl Pharmacol. 2016;303:1–10. 27105553 10.1016/j.taap.2016.04.012

[162] Chen Y, Thurman JD, Kinter LB, et al. Perspectives on using a multiplex human kidney safety biomarker panel to detect cisplatin-induced tubular toxicity in male and female Cynomolgus monkeys. Toxicol Appl Pharmacol. 2017;336:66–74. 29051111 10.1016/j.taap.2017.10.010

[163] Welty-Wolf KE, Carraway MS, Ortel TL, et al. Blockade of tissue factor-factor X binding attenuates sepsis-induced respiratory and renal failure. Am J Physiol Lung Cell Mol Physiol. 2006;290(1):L21–31. 16100288 10.1152/ajplung.00155.2005

[164] Stearns-Kurosawa DJ, Collins V, Freeman S, et al. Rescue from lethal Shiga toxin 2-induced renal failure with a cell-permeable peptide. Pediatr Nephrol. 2011;26(11):2031–2039. 21603905 10.1007/s00467-011-1913-y PMC3179571

[165] Fiedler VB, Loof I, Sander E, et al. Monoclonal antibody to tumor necrosis factor–alpha prevents lethal endotoxin sepsis in adult rhesus monkeys. J Lab Clin Med. 1992;120(4):574–588. 1402333

[166] Keshari RS, Silasi R, Popescu NI, et al. Fondaparinux pentasaccharide reduces sepsis coagulopathy and promotes survival in the baboon model of Escherichia coli sepsis. J Thromb Haemost. 2020;18(1):180–190. 31549765 10.1111/jth.14642 PMC6940562

[167] Molina L, Studenberg S, Wolberg G, et al. Efficacy of treatment with the iron (III) complex of diethylenetriamine pentaacetic acid in mice and primates inoculated with live lethal dose 100 *Escherichia coli*. J Clin Invest. 1996;98(1):192–198. 8690793 10.1172/JCI118766 PMC507416

[168] Fenhammar J, Rundgren M, Forestier J, et al. Toll-like receptor 4 inhibitor TAK-242 attenuates acute kidney injury in endotoxemic sheep. Anesthesiol J Am Soc Anesthesiol. 2011;114(5):1130–1137. 10.1097/ALN.0b013e31820b8b44 21394006

[169] Lankadeva YR, Ma S, Iguchi N, et al. Dexmedetomidine reduces norepinephrine requirements and preserves renal oxygenation and function in ovine septic acute kidney injury. Kidney Int. 2019;96(5):1150–1161. 31530477 10.1016/j.kint.2019.06.013

[170] Iguchi N, Lankadeva YR, Mori TA, et al. Furosemide reverses medullary tissue hypoxia in ovine septic acute kidney injury. Am J Physiol Regul Integr Compar Physiol. 2019;317(2):R232–r239. 10.1152/ajpregu.00371.2018 31141418

[171] Post EH, Su F, Righy Shinotsuka C, et al. Renal autoregulation in experimental septic shock and its response to vasopressin and norepinephrine administration. J Appl Physiol (1985). 2018;125:1661–1669. 10.1152/japplphysiol.00783.2017 30260750

[172] Okazaki N, Iguchi N, Evans RG, et al. Beneficial effects of vasopressin compared with norepinephrine on renal perfusion, oxygenation, and function in experimental septic acute kidney injury. Crit Care Med. 2020;48(10):e951–e958. 32931198 10.1097/CCM.0000000000004511

[173] Lankadeva YR, Kosaka J, Evans RG, et al. Urinary oxygenation as a surrogate measure of medullary oxygenation during angiotensin II therapy in septic acute kidney injury. Crit Care Med. 2018;46(1):e41–e48. 29077618 10.1097/CCM.0000000000002797

[174] Lankadeva YR, Kosaka J, Iguchi N, et al. Effects of fluid bolus therapy on renal perfusion, oxygenation, and function in early experimental septic kidney injury. Critical Care Medicine. 2019;47(1):e36–e43. 30394921 10.1097/CCM.0000000000003507

[175] Orbegozo D, Su F, Santacruz C, et al. Effects of different crystalloid solutions on hemodynamics, peripheral perfusion, and the microcirculation in experimental abdominal sepsis. Anesthesiology. 2016;125(4):744–754. 27655180 10.1097/ALN.0000000000001273

[176] Chang EI, Zárate MA, Rabaglino MB, et al. Ketamine suppresses hypoxia-induced inflammatory responses in the late-gestation ovine fetal kidney cortex. J Physiol (Lond). 2016;594(5):1295–1310. 26497972 10.1113/JP271066 PMC4771785

[177] Nilsson KF, Sandin J, Gustafsson LE, et al. The novel nitric oxide donor PDNO attenuates ovine ischemia-reperfusion induced renal failure. Intens Care Med Exp. 2017;5(1):29. 10.1186/s40635-017-0143-4 PMC5466578 28600797

[178] O'Kane D, Gibson L, May CN, et al. Zinc preconditioning protects against renal ischaemia reperfusion injury in a preclinical sheep large animal model. Biometals. 2018;31(5):821–834. 29974287 10.1007/s10534-018-0125-3

[179] Behr L, Hekmati M, Lucchini A, et al. Evaluation of the effect of autologous mesenchymal stem cell injection in a large-animal model of bilateral kidney ischaemia reperfusion injury. Cell Prolif. 2009;42(3):284–297. 19438896 10.1111/j.1365-2184.2009.00591.x PMC7662416

[180] Lankadeva YR, Cochrane AD, Marino B, et al. Strategies that improve renal medullary oxygenation during experimental cardiopulmonary bypass may mitigate postoperative acute kidney injury. Kidney Int. 2019;95(6):1338–1346. 31005272 10.1016/j.kint.2019.01.032

[181] Kampmeier T, Arnemann P, Hessler M, et al. Effects of resuscitation with human albumin 5%, hydroxyethyl starch 130/0.4 6%, or crystalloid on kidney damage in an ovine model of septic shock. Br J Anaesth. 2018;121(3):581–587. 30115256 10.1016/j.bja.2018.04.041

[182] Li N, Jin H-X, Song Z, et al. Protective effect of recombinant human brain natriuretic peptide on acute renal injury induced by endotoxin in canines. Cell Biochem Biophys. 2014;70(2):1317–1324. 24943350 10.1007/s12013-014-0057-7

[183] Rosselli DD, Mumaw JL, Dickerson V, et al. Efficacy of allogeneic mesenchymal stem cell administration in a model of acute ischemic kidney injury in cats. Res Vet Sci. 2016;108:18–24. 27663365 10.1016/j.rvsc.2016.07.003

[184] Chen Y, Harty GJ, Zheng Y, et al. CRRL269: a novel particulate guanylyl cyclase A receptor peptide activator for acute kidney injury. Circ Res. 2019;124(10):1462–1472. 30929579 10.1161/CIRCRESAHA.118.314164 PMC6512967

[185] Lee J-i, Kim M-j, Park C-s, et al. Influence of ascorbic acid on BUN, creatinine, resistive index in canine renal ischemia-reperfusion injury. J Vet Sci. 2006;7(1):79–81. 16434855 10.4142/jvs.2006.7.1.79 PMC3242091

[186] Zahran MH, Barakat N, Khater S, et al. Renoprotective effect of local sildenafil administration in renal ischaemia–reperfusion injury: a randomised controlled canine study. Arab J Urol. 2019;17(2):150–159. 31285928 10.1080/2090598X.2019.1600995 PMC6600067

[187] Lee SJ, Ryu MO, Seo MS, et al. Mesenchymal stem cells contribute to improvement of renal function in a canine kidney injury model. In Vivo. 2017;31(6):1115–1124. 29102933 10.21873/invivo.11177 PMC5756639

[188] Sekhon CS, Sekhon BK, Singh I, et al. Attenuation of renal ischemia/reperfusion injury by a triple drug combination therapy. J Nephrol. 2003;16(1):63–74. 12649537

[189] Grekas D, Kalekou H, Tourkantonis A. Effect of prostaglandin E2 (PGE2) in the prevention of acute renal failure in anesthetized dogs. In situ renal preservation. Ren Fail. 1989;11(1):27–31. 2772284 10.3109/08860228909066943

[190] Hirasawa H, Odaka M, Soeda K, et al. Experimental and clinical study on ATP-MgCl2 administration for postischemic acute renal failure. Clin Exp Dial Apheresis. 1983;7(1-2):37–47. 6603931 10.3109/08860228309076038

[191] Hardie EM, Page RL, Hoopes PJ. ATP-MgCl2 increases cisplatin toxicity in the dog and rat. J Appl Toxicol. 1992;12(5):369–375. 1447484 10.1002/jat.2550120514

[192] Margulies KB, McKinley LJ, Cavero PG, Burnett JC. Induction and prevention of radiocontrast-induced nephropathy in dogs with heart failure. Kidney Int. 1990;38(6):1101–1108. 2150085 10.1038/ki.1990.319

[193] Halpenny M, Markos F, Snow HM, et al. Effects of prophylactic fenoldopam infusion on renal blood flow and renal tubular function during acute hypovolemia in anesthetized dogs. Critic Care Med. 2001;29(4):855–860. 10.1097/00003246-200104000-00034 11373482

[194] Tuma M, Canestrini S, Alwahab Z, et al. Trauma and endothelial glycocalyx: the microcirculation helmet? Shock. 2016;46(4):352–357. 27082315 10.1097/SHK.0000000000000635

[195] Chignalia AZ, Yetimakman F, Christiaans SC, et al. The glycocalyx and trauma: a review. Shock. 2016;45(4):338–348. 26513707 10.1097/SHK.0000000000000513 PMC4792653

[196] Qureshi SH, Patel NN, Murphy GJ. Vascular endothelial cell changes in postcardiac surgery acute kidney injury. Am J Physiol Renal Physiol. 2018;314(5):F726–f735. 29357431 10.1152/ajprenal.00319.2017

[197] Gómez BI, Dubick MA, Schmidt EP, et al. Plasma and urinary glycosaminoglycans as evidence for endotheliopathy in a Swine burn model. J Surg Res. 2020;248:28–37. 31841734 10.1016/j.jss.2019.11.006

[198] Vigiola Cruz M, Carney BC, Luker JN, et al. Plasma ameliorates endothelial dysfunction in burn injury. J Surg Res. 2019;233:459–466. 30502286 10.1016/j.jss.2018.08.027

[199] Birk AV, Liu S, Soong Y, et al. The mitochondrial-targeted compound SS-31 re-energizes ischemic mitochondria by interacting with cardiolipin. J Am Soc Nephrol. 2013;24(8):1250–1261. 23813215 10.1681/ASN.2012121216 PMC3736700

[200] Landoni G, Biondi-Zoccai GG, Tumlin JA, et al. Beneficial impact of fenoldopam in critically ill patients with or at risk for acute renal failure: a meta-analysis of randomized clinical trials. Am J Kidney Dis. 2007;49(1):56–68. 17185146 10.1053/j.ajkd.2006.10.013

[201] Sun H, Xie Q, Peng Z. Does fenoldopam protect kidney in cardiac surgery? A systemic review and meta-analysis with trial sequential analysis. Shock. 2019;52(3):326–333. 30601331 10.1097/SHK.0000000000001313

[202] Yamada H, Doi K, Tsukamoto T, et al. Low-dose atrial natriuretic peptide for prevention or treatment of acute kidney injury: a systematic review and meta-analysis. Crit Care. 2019;23(1):41. 30744687 10.1186/s13054-019-2330-z PMC6371622

[203] Tögel FE, Westenfelder C. Mesenchymal stem cells: a new therapeutic tool for AKI. Nat Rev Nephrol. 2010;6(3):179–183. 20186233 10.1038/nrneph.2009.229

[204] Chung BH. Use of mesenchymal stem cells for chronic kidney disease. Kidney Res Clin Pract. 2019;38(2):131–134. 31189218 10.23876/j.krcp.19.051 PMC6577207

[205] Nutton V. Portraits of science. Logic, learning, and experimental medicine. Science. 2002;295(5556):800–801. 11823624 10.1126/science.1066244

[206] Giraud S, Favreau F, Chatauret N, et al. Contribution of large pig for renal ischemia-reperfusion and transplantation studies: the preclinical model. J Biomed Biotechnol. 2011;2011:532127. 21403881 10.1155/2011/532127 PMC3051176

[207] Swindle MM, Makin A, Herron AJ, Clubb FJ, Jr, et al. Swine as models in biomedical research and toxicology testing. Vet Pathol. 2012;49(2):344–356. 21441112 10.1177/0300985811402846

[208] Maurya H, Kumar T, Kumar S. Anatomical and physiological similarities of kidney in different experimental animals used for basic studies. J Clin Exp Nephrol. 2018;3:9.

[209] Fabian Alejandro G, Luis Ernesto B, Hernando Yesid E. Morphological characterization of the renal arteries in the pig. Comparative analysis with the human. Int J Morphol. 2017;35(1):319–324.

[210] Aleksiewicz R, Lutnicki K, Bojarski M, et al. Haemodynamics imaging of swine segmental kidney artery using duplex doppler technique. J Vet Res. 2019;63(2):259–265. 31276066 10.2478/jvetres-2019-0036 PMC6598193

[211] Kazzaz D, Shanklin WM. Comparative anatomy of the superficial vessels of the mammalian kidney demonstrated by plastic (vinyl acetate) injections and corrosion. J Anat. 1951;85(2):163–165. 14824029 PMC1273595

[212] Dhondt L, Croubels S, De Paepe P, et al. Conventional pig as animal model for human renal drug excretion processes: unravelling the porcine renal function by use of a cocktail of exogenous markers. Front Pharmacol. 2020;11:883. 32595506 10.3389/fphar.2020.00883 PMC7303324

[213] Ekser B, Rigotti P, Gridelli B, et al. Xenotransplantation of solid organs in the pig-to-primate model. Transpl Immunol. 2009;21(2):87–92. 18955143 10.1016/j.trim.2008.10.005

[214] Inowa T, Hishikawa K, Takeuchi T, et al. Isolation and potential existence of side population cells in adult human kidney. Int J Urol. 2008;15(3):272–274. 18304230 10.1111/j.1442-2042.2007.01984.x

[215] Burmeister DM, McIntyre MK, Montgomery RK, et al. Isolation and characterization of multipotent CD24+ cells from the renal papilla of swine. Front Med (Lausanne). 2018;5:250. 30283781 10.3389/fmed.2018.00250 PMC6156461

[216] Dekel B, Burakova T, Arditti FD, et al. Human and porcine early kidney precursors as a new source for transplantation. Nat Med. 2003;9(1):53–60. 12496960 10.1038/nm812

[217] Sleeman P, Patel NN, Lin H, et al. High fat feeding promotes obesity and renal inflammation and protects against post cardiopulmonary bypass acute kidney injury in swine. Crit Care. 2013;17(5):R262. 24172587 10.1186/cc13092 PMC4056797

[218] Danziger J, Chen K, Lee J, et al. Obesity, acute kidney injury, and mortality in critical illness. Critic Care Med. 2016;44(2):328. 10.1097/CCM.0000000000001398 PMC4715729 26496453

[219] Alidori S, Akhavein N, Thorek DLJ, et al. Targeted fibrillar nanocarbon RNAi treatment of acute kidney injury. Sci Transl Med. 2016;8(331):331ra39. 10.1126/scitranslmed.aac9647 PMC5004247 27009268

[220] Yang C, Li L, Xue Y, et al. Innate immunity activation involved in unprotected porcine auto-transplant kidneys preserved by naked caspase-3 siRNA. J Transl Med. 2013;11(1):210. 24034868 10.1186/1479-5876-11-210 PMC3847504

[221] Yang C, Yang B. siRNA-induced RNAi therapy in acute kidney injury. RNA Interf. 2016;223.

[222] Thompson JD, Kornbrust DJ, Foy JW, et al. Toxicological and pharmacokinetic properties of chemically modified siRNAs targeting p53 RNA following intravenous administration. Nucleic Acid Ther. 2012;22(4):255–264. 22913596 10.1089/nat.2012.0371 PMC3426203

[223] Fan P-C, Chen C-C, Chen Y-C, et al. MicroRNAs in acute kidney injury. Hum Genomics. 2016;10(1):29. 27608623 10.1186/s40246-016-0085-z PMC5016954

[224] Han SJ, Williams RM, D’Agati V, et al. Selective nanoparticle-mediated targeting of renal tubular Toll-like receptor 9 attenuates ischemic acute kidney injury. Kidney Int. 2020;98(1):76–87. 32386967 10.1016/j.kint.2020.01.036 PMC7872414

[225] Haas M, Kluppel ACA, Wartna ES, et al. Drug-targeting to the kidney: renal delivery and degradation of a naproxen-lysozyme conjugate in vivo. Kidney Int. 1997;52(6):1693–1699. 9407519 10.1038/ki.1997.504

[226] Liu KD, Humphreys BD, Endre ZH. The ten barriers for translation of animal data on AKI to the clinical setting. Intensive Care Med. 2017;43(6):898–900. 28451772 10.1007/s00134-017-4810-4 PMC5515621

